# Long-term health of children following the Eyjafjallajökull volcanic eruption: a prospective cohort study

**DOI:** 10.1080/20008198.2018.1442601

**Published:** 2018-03-05

**Authors:** Heidrun Hlodversdottir, Harpa Thorsteinsdottir, Edda Bjork Thordardottir, Urdur Njardvik, Gudrun Petursdottir, Arna Hauksdottir

**Affiliations:** ^a^ Centre of Public Health Sciences, University of Iceland, Reykjavık, Iceland; ^b^ Faculty of Psychology, School of Health Sciences, University of Iceland, Reykjavik, Iceland; ^c^ The Institute of Sustainability Studies, University of Iceland, Reykjavik, Iceland

**Keywords:** Volcano eruption, disaster, children, physical health, mental health, prospective cohort study, Erupción volcánica, desastre, niños, salud física, salud mental, estudio de cohorte prospectivo, 火山爆发, 灾难, 儿童, 身体健康, 精神健康, 前瞻队列研究, • Studies on the effects of volcanic eruptions on children’s health are sparse, especially regarding potential health effects beyond the first year.• Adverse physical and mental health problems experienced by the children exposed to the eruption seem to persist for up to a three-year period post-disaster.• An important strength of this study is that it includes a large population-based cohort exposed to the Eyjafjallajökull eruption and a matched cohort from a non-exposed population all contacted at two points in time with high response rate.

## Abstract

**Background**: More than 500 million people worldwide live within exposure range of an active volcano and children are a vulnerable subgroup of such exposed populations. However, studies on the effects of volcanic eruptions on children’s health beyond the first year are sparse.

**Objective**: To examine the effect of the 2010 Eyjafjallajökull eruption on physical and mental health symptoms among exposed children in 2010 and 2013 and to identify potential predictive factors for symptoms.

**Method**: In a population-based prospective cohort study, data was collected on the adult population (*N* = 1615) exposed to the 2010 Eyjafjallajökull eruption and a non-exposed group (*N* = 697). The exposed group was further divided according to exposure level. All participants answered questionnaires assessing their children´s and their own perceived health status in 2010 and 2013.

**Results**: In 2010, exposed children were more likely than non-exposed children to experience respiratory symptoms (medium exposed OR 1.47; 95% CI 1.07–2.03; high exposed OR 1.52; 95% CI 1.03–2.24) and anxiety/worries (medium exposed OR 2.39; 95% CI 1.67–3.45; high exposed OR 2.77; 95% CI 1.81–4.27). Both genders had an increased risk of symptoms of anxiety/worries but only exposed boys were at increased risk of experiencing headaches and sleep disturbances compared to non-exposed boys. Within the exposed group, children whose homes were damaged were at increased risk of experiencing anxiety/worries (OR 1.62; 95% CI 1.13–2.32) and depressed mood (OR 1.55; 95% CI 1.07–2.24) than children whose homes were not damaged. Among exposed children, no significant decrease of symptoms was detected between 2010 and 2013.

**Conclusions**: Adverse physical and mental health problems experienced by the children exposed to the eruption seem to persist for up to a three-year period post-disaster. These results underline the importance of appropriate follow-up for children after a natural disaster.

## Abstract (es)

**Planteamiento**: Más de 500 millones de personas en todo el mundo viven dentro del área de exposición de un volcán activo y los niños son un subgrupo vulnerable de dichas poblaciones que se encuentran expuestas. Sin embargo, hasta la fecha, los estudios sobre los efectos de las erupciones volcánicas en la salud de los niños más allá del primer año son escasos.

**Objetivo**: Examinar el efecto de la erupción del Eyjafjallajökull en 2010 en los síntomas de salud física y mental entre niños expuestos en 2010 y 2013 e identificar los posibles factores predictivos de los síntomas.

**Método**: En un estudio de cohortes prospectivo basado en la población, se recopiló información sobre la población adulta (*N* = 1615) que estuvo expuesta a la erupción del Eyjafjallajökull en 2010 y un grupo que no estuvo expuesto (*N* = 697). El grupo expuesto se dividió además según el nivel de exposición. Todos los participantes respondieron cuestionarios que evaluaban el estado de salud que percibían tanto sus hijos como de sí mismos en 2010 y 2013.

**Resultados**: En 2010, los niños expuestos tenían más probabilidades que los niños no expuestos de experimentar síntomas respiratorios (OR exposición media 1.47, IC 95% 1.07-2.03, OR exposición elevada 1.52, IC 95% 1.03-2.24) y ansiedad/preocupaciones (OR exposición media 2,39; IC del 95%: 1,67 a 3,45; OR exposición elevada, 77; IC del 95%: 1,81 a 4,27). Ambos sexos tenían un mayor riesgo de síntomas de ansiedad/preocupaciones, pero solo los niños expuestos tenían un mayor riesgo de experimentar dolores de cabeza y trastornos del sueño en comparación con los niños no expuestos. Dentro del grupo expuesto, los niños cuyas casas sufrieron daños tenían un mayor riesgo de experimentar ansiedad/preocupaciones (OR 1.62, IC 95% 1.13-2.32) y estado de ánimo deprimido (OR 1.55, IC 95% 1.07-2.24) que los niños cuyas casas no estaban dañado. Entre los niños expuestos, no se detectó una disminución significativa de los síntomas entre 2010 y 2013.

**Conclusiones**: Los problemas físicos y mentales adversos experimentados por los niños expuestos a la erupción parecen persistir durante un período de hasta tres años después del desastre. Estos resultados subrayan la importancia de un seguimiento adecuado para los niños y la identificación de los posibles grupos de riesgo después de un desastre natural.

## Abstract (zh)

**背景**: 全世界超过5亿人暴露在活火山范围内，其中儿童属于敏感人群。但是目前为止，关于火山爆发对一年后儿童健康的影响的研究还很少见。

**目标**: 考察埃亚菲亚德拉火山在2010年爆发后对暴露儿童在2010年和2013年的身心健康的影响，并识别症状的预测因子。

**方法**: 在一个基于全人群的前瞻队列研究，在经历2010年埃亚菲亚德拉火山爆发的成人群体（*N*=1615）和没有经历的群体（*N*=697）中采集数据。暴露组进一步根据暴露程度细分。所有的参与者都回答了关于他们子女和他们自身在2010年和2013年的健康状态。

**结果**: 2010年，经历火山爆发的和没有经历的儿童相比更有可能出现呼吸系统症状（中度暴露 OR 1.47; 95% CI 1.07-2.03; 高度暴露 OR 1.52; 95% CI 1.03-2.24)和焦虑/担忧(中度暴露 OR 2.39; 95% CI 1.67-3.45; 高度暴露 OR 2.77; 95% CI 1.81-4.27)。和非暴露组相比，暴露组的男女性别都有焦虑/担忧症状增多的风险，但只有暴露组的男孩有更多的头痛症状和睡眠问题。在暴露组内，住房被毁坏的比住房没有被毁坏的儿童有更多风险出现焦虑/担忧症状 (OR 1.62; 95% CI 1.13-2.32) 和抑郁情绪(OR 1.55; 95% CI 1.07-2.24) 。但在暴露组儿童中，从2010年到2013年没有显著的症状减轻。

**结论**: 暴露于火山爆发的儿童出现的身心健康问题，在灾害后似乎持续了三年时间。这些结果强调了对儿童进行恰当追踪研究的重要性，和识别自然灾害后的可能风险群体的重要性。

## 1. Introduction

More than 500 million people worldwide live within the exposure range of an active volcano, which would subject them to increased health hazards if an eruption took place (Small & Naumann, 2001; Tilling & Lipman, 1993). Children are a vulnerable subgroup of such exposed populations. However, to date, studies on the effects of volcanic eruptions on children’s health are sparse, especially regarding potential health effects beyond the first year.

In the spring of 2010, an eruption began in the Eyjafjallajökull volcano in Iceland, lasting for almost six weeks. Direct ash fall from the eruption was estimated to be around 250 million tons. Due to prevailing weather conditions the plume of volcanic ash spread mainly southwards to Europe. Rural regions south and south-east of the volcano were heavily exposed to continuous ash fall for six weeks with frequent re-suspension for many months to follow (Thorsteinsson, Jóhannsson, Stohl, & Kristiansen, 2012). The continuous ashfall darkened the environment to the point of turning daylight into night and, in addition, glacier flooding, heavy lightning strikes, loud volcanic sound blasts and lava flows impacted the daily life of the exposed residents. This raised concerns of health hazards, with adults being particularly worried about children´s health (Bird & Gisladottir, 2012).

Previous studies have reported adverse respiratory symptoms following exposure to volcanic ash, both among children (Forbes, Jarvis, Potts, & Baxter, 2003; Naumova et al., 2007) and adults (Buist, Vollmer, Johnson, Bernstein, & Mccamant, 1986; Carlsen et al., 2012, 2012; Fano et al., 2006; Horton & McCaldin, 1964; Horwell & Baxter, 2006; Rojas-Ramos et al., 2001). Respiratory symptoms have also been found to be more common among both children and adults in high exposed areas, compared to non-exposed and low exposed areas (Carlsen et al., 2012; Forbes et al., 2003). In addition, long-term respiratory symptoms have also been detected among adults after the Eyjafjallajökull eruption, with the risk of certain respiratory symptoms increasing over time. Three to four years after the eruption, symptoms still reflected the severity of exposure (Hlodversdottir, Petursdottir, Carlsen, Gislason, & Hauksdottir, 2016).

In addition, exposure to natural disasters has been associated with adverse effects on mental health. The majority of studies on children’s well-being following natural disasters have focused on post-traumatic stress disorder (PTSD) (Bokszczanin, 2007; Catani, Jacob, Schauer, Kohila, & Neuner, 2008; Fan, Zhang, Yang, Mo, & Liu, 2011; Goenjian et al., 2011; John, Russell, & Russell, 2007; Lai, Kelley, Harrison, Thompson, & Self-Brown, 2015; Self-Brown, Lai, Thompson, McGill, & Kelley, 2013; Stanke, Murray, Amlot, Nurse, & Williams, 2012). Depression and anxiety have also been reported among children post-disaster, and are often comorbid with PTSD (La Greca, Silverman, Lai, & Jaccard, 2010; Lai, La Greca, Auslander, & Short, 2013; Swenson et al., 1996). Other potential post-disaster psychological sequelae have received less attention, such as children’s behaviour problems, substance abuse and sleep disturbances (Pfefferbaum, Jacobs, Griffin, & Houston, 2015). The effect of disaster exposure on behavioural problems has led to inconsistent results. Swenson et al. (1996) reported a short-term increase in generally defined behaviour problems post-disaster among young children; Fujiwara et al., 2014 reported 26% of young children had behaviour problems two years post-disaster. Other studies have indicated a delayed onset of symptoms with a rise in the rate of behaviour problems 1–2 years post-disaster (Pine & Cohen, 2002). To date, the effect of trauma on sleep disturbances among children had mainly been researched in the context of PTSD (Charuvastra & Cloitre, 2009). However, emerging research indicates that sleep disruptions may be a distinct disorder after trauma (Mysliwiec et al., 2018) .

With regard to predictive factors, some studies have found girls to be more likely than boys to show symptoms of post-traumatic stress following disasters (Garrison et al., 1995; Giannopoulou et al., 2006; John et al., 2007; Lai et al., 2013), while others have found no gender specific effects (Catani et al., 2008). In addition, research has indicated that experiencing secondary life stressors such as loss of home, having to change schools and shifts in parental employment and finances should increase the risk of adverse mental health outcomes among children post-disaster (Fan et al., 2011; La Greca et al., 2010; Silverman, 2002). Other factors have also been associated with negative effects on children’s mental health, such as parental distress, negative parenting practices and lack of peer support (Kelley et al., 2010; Self-Brown et al., 2013).

Usually, psychological symptoms peak during the first year post-disaster (Eksi & Braun, 2009; Fan, Long, Zhou, Zheng, & Liu, 2015; Piyasil et al., 2011), but some studies indicate that symptoms do not significantly decrease over time (Jia et al., 2013; Thienkrua et al., 2006; Weems et al., 2010). Long-term studies have even found an increase in psychological symptoms years after the disaster (Shaw, Applegate, & Schorr, 1996; Ye, Fan, Li, & Han, 2014). Findings from studies on the long-term sequelae of psychological symptoms of children and adolescents following disasters are therefore mixed. More information is needed on more diverse outcomes than previously studied and on moderating and predictive factors of psychological impacts that natural disasters may have on children in order to improve preparedness and interventions for this vulnerable group.

This population-based study was carried out at two points in time following the 2010 Eyjafjallajökull volcano eruption: 6–9 months and three years after the eruption ended. The overall aim was to examine the effects of the eruption on physical and mental symptoms among children living in the exposed area and how symptoms develop over a three-year period. In addition, we aimed to assess factors potentially associated with children’s symptoms, level of exposure, gender, age, damages to home and parents’ well-being.

## 2. Methods

### 2.1. Study area

**Figure 1.**
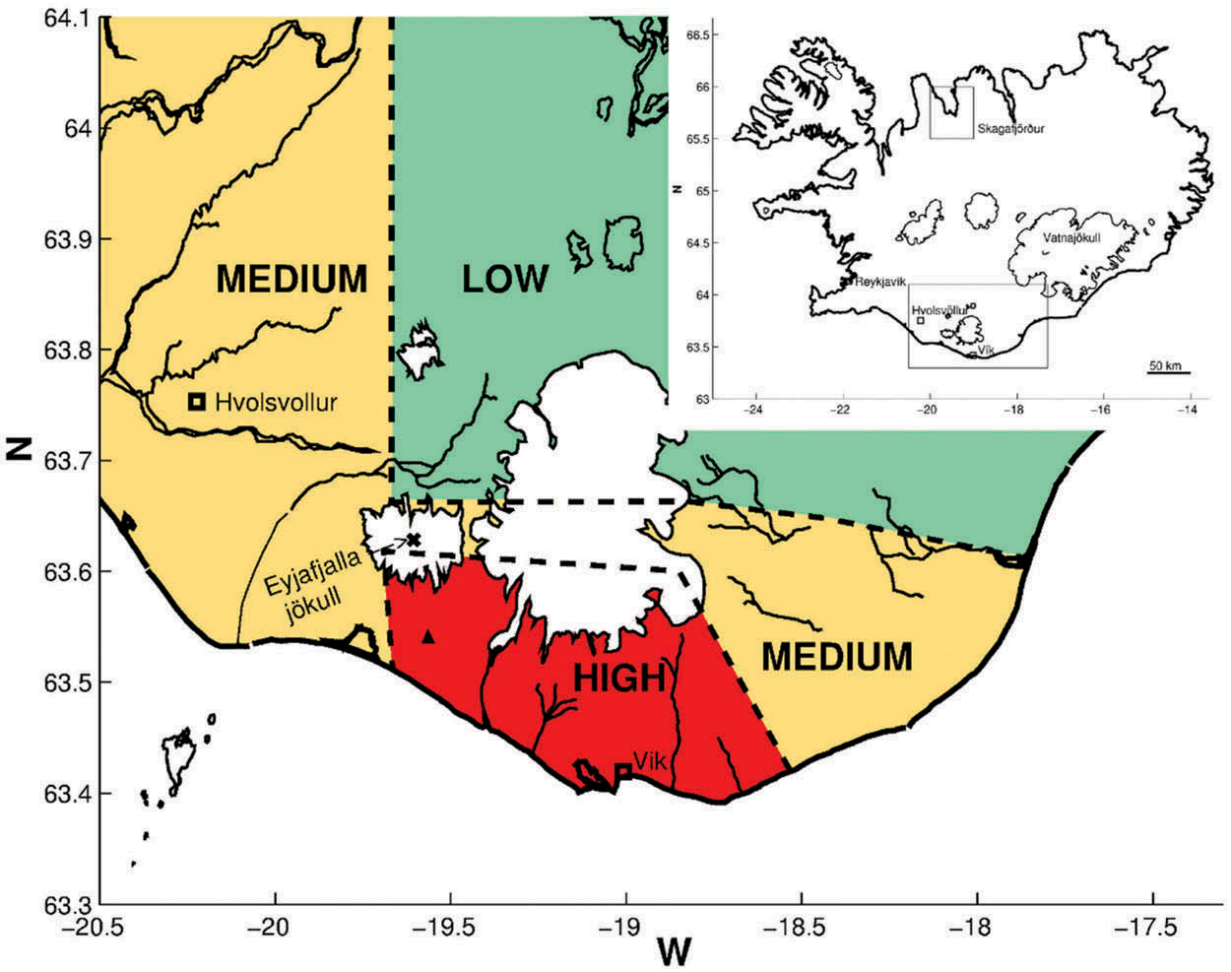
Map of Iceland and study areas (as defined in Carlsen et al., 2012) Inserted map of Iceland in the right corner shows the location of Skagafjörður in Northern Iceland (non-exposed area) and the exposed area in South Iceland. The larger map shows the exposed area with Eyjafjallajökull marked as X, the site of the measuring station with a ▲ and the exposed areas divided to low, medium and high exposed areas.

The exposed area in southern Iceland (see Figure 1) was divided into three levels of exposure, depending on the intensity of ash fall as measured and modelled by the Environment Agency of Iceland, which monitored concentrations of inhalable particulate matter (PM10) in several locations in the study area. The exposed area was divided into low, medium and high exposure based on satellite images of the eruption plume, information about the emission intensity and observation of ash deposits on the ground (Carlsen et al., 2012). For comparison, a non-exposed group was defined in a rural non-exposed area in northern Iceland.

### 2.2. Study population

The health status of the children in this study was acquired through report of the children’s parents. Questionnaires were sent out to the exposed study population (*N* = 1615), consisting of all adult residents (age 18–80) who were located in the area at the time of the eruption, spoke Icelandic fluently and could be reached at the time of the study in the municipalities closest to the Eyjafjallajökull volcano (pre-defined by six postal codes) and identified in the population-based registry in Iceland (Registers Iceland). A total of 1148 adult participants answered the questionnaire. The comparison group included a random sample from an adult non-exposed population in North Iceland (*N* = 697) matched to the exposed group with regard to age, gender and urban/rural habitation. A total of 510 adult participants sent in the questionnaire. Our analyses included all participants that answered questions on their children’s health, in total, parents of 781 exposed children and 362 non-exposed children in 2010. Three years later, those who had participated in 2010 were contacted again. Fifty-two members of the exposed adults group and 35 of the non-exposed adults group could not be found in the registers or had moved abroad, leaving the study population with 1096 adult participants from the exposed area and 475 adult participants from the non-exposed area. In 2013, in total, parents of 475 exposed children and 189 non-exposed children were included in our analysis for the present study.

### 2.3. Data collection

In the 2010 study, 6–9 months after the eruption, participants in the exposed group received an invitation to participate in the study as well as information about the study. Few days later they were contacted by telephone and asked whether they wanted to participate and given the choice of filling out an online or paper questionnaire. Questionnaires or email invitations were subsequently sent to the participants and a week later a combined thank you/reminder card was sent out. Participants were reminded again by telephone if needed. A similar protocol was used for the non-comparison group, except that the introductory letter stated that a questionnaire would be sent within a week, unless they declined to participate. The participants choice in 2010 determined the kind of questionnaire they received in the latter study in 2013. In 2010, questionnaires were sent out between November 2010 and February 2011. At time point two, questionnaires were sent out between December 2013 and February 2014. Every participant received a thank you/reminder card a few weeks after the questionnaires had been sent out. Those who had not replied within a certain time were reminded to do so by email and/or by phone.

The questionnaires did not contain information that revealed the identity of the respondent, but had a running number which could be linked to the person’s ID number through a list which was kept separately and securely to enable the linkage between the 2010 and 2013 datasets. The study was approved by The Icelandic Data Protection Authority (no. S4878/2010) and The Science Bioethics Committee (no. VSNb2010080002/03.7) in Iceland. All participants gave informed consent.

### 2.4. Measurements

The questionnaires included a list of demographic questions as well as detailed questions on physical and mental symptoms experienced by adult participants following the eruption. They also contained questions about the participants’ children, regarding the child’s age and gender and whether the parents/custodians had observed any of the following seven symptoms in the child in the past month: (a) respiratory symptoms, (b) stomach pain or nausea, (c) headache(s), (d) sleep disturbances, (e) anxiety or worries, (f) depressed mood and (g) behavioural problems. Each item was graded on a four-point scale with response options as follows: (1) no; (2) yes, a little; (3) yes, somewhat; and (4) yes, much. For the statistical analyses, scores were computed as binary, symptomology present or absent. The options (2) yes, a little, (3) yes, somewhat and (4) yes, much was considered as symptom ‘present’. The (1) ‘no’ option was considered as symptom ‘absent’. The questionnaires included questions for up to three children living at home. If more children were living at home, the respondents were encouraged to begin with the youngest child and answer for up to three children and add information on older children in an open-ended fashion.

Psychological distress of adult participants was measured with the General Health Questionnaire 12-item version (GHQ-12), a well-known and widely used instrument (Hankins, 2008). The GHQ-12 is a self-reported screening tool that consists of 12 items, used to assess the severity of mental distress over the past few weeks. The GHQ-12 has a four-point response scale where scores are coded on a bimodal scale for symptom present ‘not at all’ (0), ‘same as usual’ (0), ‘rather more than usual’ (1) and ‘much more than usual’ (1), resulting in a possible score range from 0 to 12. A binary cut-off score of >2 was used in the current study which is indicative of experiencing more mental distress than usual (Goldberg et al., 1997).

Regarding material damages, the questionnaires included the questions: ‘Have you experienced damages as a result of the volcanic eruption to the following: (a) farm/domestic animals, (b) residence, (c) barns/sheds, (d) other nearby constructions (e.g. garage or fences), (e) yard and (f) farmland’. For this study, the option (b) ‘residence’ was considered as having experienced damages. Response options were categorized into (1) no (no, not relevant, does not apply) and (2) yes (a little or a lot). For the statistical analyses, answers were computed as binary, experiencing damages to house (yes) or not (no).

### 2.5. Statistical analysis

Demographic characteristics were compared between the exposed and the non-exposed populations from 2010 using the χ^2^ test. In addition, logistic regression analysis was used to calculate ORs and 95% CIs for the association between children´s symptoms and possible predictive factors associated with residence in (1) the low, medium, high exposure areas and non-exposed area, (2) the low, medium and high-exposed groups within the exposed area and in (3) the exposed area in 2010 and 2013. These models were adjusted for a priori selected variables; possible confounders were age and gender of the children. Results were considered statistically significant when *p* values were ≤ .05 or the CIs did not include 1.0. Statistical analysis was performed with R: the R Project for Statistical Computing (RC-Team, 2012).

## 3. Results

### 3.1. Participants

In 2010, response rate was 71% (1148/1615) for the adult exposed population and 73% for the adult non-exposed population (510/697). Of 1148 participants in the exposed area, 433 had a child or children living at home; in total 781 children under 18 years of age. Of the 510 adult participants from the non-exposed area, 200 had one or more children living at home, in total 372 children. In 2010, the exposed and non-exposed adult populations were similar regarding age, gender, education, marital status and smoking status. Distribution of gender and age of participants’ children was also similar in the exposed and non-exposed populations (all *p*-values > .05) (see Table 1). In 2013, valid questionnaires were received from 874 of 1096 in the exposed population (80%) where 287 had a child or children living at home, in total 475 children living at home. Valid questionnaires were received from 381 of 475 (80%) in the non-exposed population in 2013, where 123 had a child or children living at home, in total 189 children living at home. The exposed group of adults differed statistically significantly between the years 2010 and 2013 regarding age, education, marital status household size, occupational status and financial status, but was similar regarding gender and smoking status (data not shown).

**Table 1.** Demographic characteristics of participants in a study on the health effects of children following the volcanic eruption in Eyjafjallajökull in Iceland 2010 (classified by exposure).

	Non-exposed	Exposed	
	% (*n*/*N*)	% (*n*/*N*)	*p*-value*
Participants answering questions on children’ well-being	39.2 (200/510)	37.8 (433/1146)	
Participants characteristics			
Gender			.39
Male	51.0 (102/200)	47.3 (205/433)	
Female	49.0 (98/200)	52.7 (228/433)	
Age Categories			.39
18–23	5.0 (10/200)	3.9 (17/433)	
24–30	13.5 (27/200)	9.5 (41/433)	
31–40	30.0 (60/200)	31.2 (135/433)	
41–50	37.0 (74/200)	40.6 (176/433)	
51–60	13.0 (26/200)	11.3 (49/433)	
61–70	1.0 (2/200)	3.2 (14/433)	
71–80	0.5 (1/200)	0.2 (1/433)	
Education			.17
Primary education or less	29.5 (59/200)	29.0 (126/433)	
Secondary education	38.0 (76/200)	40.4 (175/433)	
Professional or university education	31.5 (63/200)	27.0 (117/433)	
Other education*	1.0 (2/200)	3.5 (15/433)	
Marital status			.97
Married or cohabiting	90.0 (180/200)	90.9 (394/433)	
Single, divorced or widow/widower	6.5 (13/200)	5.9 (26/433)	
Relationship-no cohabitation	3.5 (7/200)	3.0 (13/433)	
Total number of children	*N* = 372	*N* = 781	
Children´s characteristics			
Gender			.34
Boy	50.0 (186/372)	53.0 (414/781)	
Girl	50.0 (186/372)	47.0 (367/781)	
Age			.73
0–5 years	29.0 (105/362)	27.8 (216/777)	
6–11 years	34.3 (124/362)	36.7 (285/777)	
12–18 years	36.7 (133/362)	35.5 (276/777)	

* *P*-value < .05.

### 3.2. Children’s symptoms in 2010 by level of exposure

In 2010, using the non-exposed children as a reference, the following statistically significant differences were observed when adjusting for age and gender (see Table 2 for details): higher likelihood of respiratory symptoms (medium exposed OR 1.47; 95% CI 1.07 to 2.03; high exposed OR 1.52; 95% CI 1.03 to 2.24), greater anxiety or worries (medium exposed OR 2.39; 95% CI 1.67 to 3.45; high exposed OR 2.77; 95% CI 1.81 to 4.27), more headache(s) (medium exposed OR 1.91; 95% 1.37 to 2.68; high exposed OR 2.19; 95% CI 1.46 to 3.28) and more sleep disturbances (high exposed OR 1.65; 95% CI 1.10 to 2.46). When using low exposed children as a reference, statistical significant difference was only detected in sleep disturbances among high exposed children (OR 1.95; 95% CI 1.02 to 3.95).

**Table 2.** Risk of symptoms in children in a population exposed to the Eyjafjallajökull volcanic eruption by residence exposure level in 2010.

	Non-exposed 2010 (*n* = 372)		Low exposure 2010* (*n* = 78)		Medium exposure 2010* (*n* = 485)		High exposure 2010* (*n* = 218)	
	OR (95% CI)^†^	% (*n*/*N*)	OR (95% CI)^†^	% (*n*/*N*)	OR (95% CI)^†^	% (*n*/*N*)	OR (95% CI)^†^	% (*n*/*N*)
Respiratory symptoms	1 (reference)	23.4 (86/367)	0.85 (0.44 to 1.55)	19.5 (15/77)	1.47 (1.07 to 2.03)	31.4 (150/477)	1.52 (1.03 to 2.24)	31.9 (67/210
			1 (reference)		1.73 (0.97 to 3.27)		1.79 (0.96 to 3.50)	
Stomach pain/nausea	1 (reference)	28.3 (104/367)	0.72 (0.39 to 1.28)	23.1 (18/78)	1.02 (0.75 to 1.39)	29.1 (139/478)	1.05 (0.71 to 1.53)	28.9 (61/211)
			1 (reference)		1.41 (0.81 to 2.58)		1.45 (0.79 to 2.75)	
Anxiety/worries	1 (reference)	14.8 (54/364)	1.47 (0.75 to 2.76)	19.5 (15/77)	2.39 (1.67 to 3.45)	28.5 (136/478)	2.77 (1.81 to 4.27)	31.6 (66/209)
			1 (reference)		1.63 (0.90 to 3.10)		1.89 (1.00 to 3.74)	
Behavioural problems	1 (reference)	18.5 (67/363)	0.69 (0.32 to 1.37)	13.0 (10/77)	0.96 (0.67 to 1.38)	17.9 (85/474)	1.27 (0.83 to 1.94)	23.2 (49/211)
			1 (reference)		1.39 (0.71 to 2.99)		1.84 (0.91 to 4.07)	
Headache(s)	1 (reference)	20.9 (76/364)	1.18 (0.63 to 2.16)	23.1 (18/78)	1.91 (1.37 to 2.68)	31.0 (147/474)	2.19 (1.46 to 3.28)	33.6 (71/211)
			1 (reference)		1.61 (0.91 to 2.98)		1.85 (1.00 to 3.55)	
Depressed mood	1 (reference)	22.0 (80/364)	0.83 (0.43 to 1.53)	18.2 (14/77)	1.14 (0.82 to 1.60)	23.8 (114/478)	1.35 (0.90 to 2.00)	27.6 (59/214)
			1 (reference)		1.38 (0.76 to 2.66)		1.62 (0.86 to 3.24)	
Sleep disturbances	1 (reference)	21.0 (76/362)	0.85 (0.42 to 1.58)	16.9 (13/77)	1.10 (0.80 to 1.55)	22.9 (109/477)	1.65 (1.10 to 2.46)	29.0 (61/210)
			1 (reference)		1.29 (0.70 to 2.55)		1.95 (1.02 to 3.95)	

*Regions are seen in Figure 1

^†^OR and 95% CI from multivariate logistic regression adjusted for age and gender.

Gender-specific analyses revealed that exposed boys were more likely than non-exposed boys to experience: sleep disturbances (OR 1.62; 95% CI 1.04 to 2.59), anxiety or worries (OR 2.30; 95% CI 1.43 to 3.82) and headache(s) (OR 2.48; 95% CI 1.57 to 4.02) (adjusted for age). Exposed girls were more likely to experience anxiety or worries (OR 2.50; 95% CI 1.47 to 4.07) than non-exposed girls, when adjusted for their age.

### 3.3. Damages to home and children’s symptoms

Children whose homes had been damaged following the eruption were at increased risk for anxiety or worries (OR 1.62; 95% CI 1.13 to 2.32) and depressed mood (OR 1.55; 95% CI 1.07 to 2.24), but not stomach pain or nausea, headache(s), sleep disturbances and behavioural problems.

### 3.4. Parental mental health and children’s symptoms

We also examined children´s symptoms by parental psychological morbidity as measured by the General Health Questionnaire (see Table 3). No statistical significant difference was detected for the symptoms (respiratory, stomach pain/nausea, anxiety/worries, headache(s), depressed mood and sleep disturbances) between non-exposed and exposed children whose parents reported symptoms of psychological morbidity. Although not statistically significant, we observed a tendency for increase for anxiety or worries (OR 3.49; 95% CI 0.85 to 18.38) and headache(s) (OR 4.40; 95% CI 0.88 to 31.48).

**Table 3.** Children´s symptoms by parental psychological morbidity.

	Non-exposed	Exposed	
	% (*n*/*N*)	% (*n*/*N*)	OR (95% CI)^†^
Children’s symptoms			
**Anxiety/worries**			
Parental psychological morbidity φ (GHQ score ˃ 2)	16.7 (3/18)	47.5 (19/40)	3.49 (0.85 to 18.38)
**Headache(s)**			
Parental psychological morbidity (GHQ score ˃ 2)	16.7 (3/18)	45.0 (18/40)	4.40 (0.88 to 31.48)
**Depressed mood**			
Parental psychological morbidity (GHQ score > 2)	38.9 (7/18)	37.5 (15/40)	0.68 (0.20 to 2.34)
**Sleep disturbances**			
Parental psychological morbidity (GHQ score > 2)	17.6 (3/17)	32.5 (13/40)	1.98 (0.51 to 9.80)
**Stomach pain/nausea**			
Parental psychological morbidity (GHQ score > 2)	27.8 (5/18)	25.0 (10/40)	0.81 (0.23 to 3.11)
**Behavioral problems**			
Parental psychological morbidity (GHQ score > 2)	41.1 (7/17)	23.7 (9/38)	0.33 (0.09 to 1.21)

^†^OR and 95% CI from multivariate logistic regression adjusted for age and gender

φ Parental psychological morbidity was measured with the GHQ-12 (General Health Questionnaire) referring to ‘the previous weeks’, using a binary cut-off score of > 2.

### 3.5. Development of children’s symptoms between 2010 and 2013

Table 4 presents the development of exposed children’s symptoms in 2010 and 2013 where no statistical significant differences were detected in respiratory symptoms, stomach pain or nausea, anxiety or worries, behavioural problem, headache(s), depressed mood, sleep disturbances between those two time points.

**Table 4.** Risk of symptoms in children in a population exposed to the Eyjafjallajökull volcanic eruption.

	Exposed 2010	Exposed 2013	
	(*n* = 781)	(*n* = 475)	
	% (*n*/*N*)	% (*n*/*N*)	OR (95% CI)^†^
Respiratory symptoms	30.4 (232/764)	23.8 (112/470)	0.97 (0.85 to 1.10)
Stomach pain/nausea	28.4 (218/767)	26.3 (122/463)	0.98 (0.86 to 1.11)
Headache(s)	30.9 (236/763)	26.4 (123/466)	0.95 (0.84 to 1.09)
Anxiety/worries	28.4 (217/764)	25.9 (120/464)	0.98 (0.87 to 1.11)
Behavioural problems	18.9 (144/762)	18.2 (83/455)	1.05 (0.90 to 1.22)
Depressed mood	24.3 (187/769)	25.2 (117/465)	1.09 (0.95 to 1.24)
Sleep disturbances	24.0 (183/764)	21.9 (101/461)	1.00 (0.87 to 1.16)

^†^OR and 95% CI from multivariate logistic regression adjusted for age and gender.

### 3.6. Children’s symptoms in 2013 by level of exposure

In 2013, using the non-exposed children as a reference, no statistically significant differences were found between levels of exposure for any the symptoms (see Table 5). However, when using low exposed children as a reference in 2013 the following statistical significant differences were detected; depressed mood (medium exposed OR 2.82; 95% CI 1.24 to 7.63) and sleep disturbances (high exposed OR 2.82; 95% CI 1.10 to 8.73).

**Table 5.** Risk of symptoms in children in a population exposed to the Eyjafjallajökull volcanic eruption by residence exposure level in 2013.

	Non-exposed 2013 (*n* = 189)		Low exposure 2013* (*n* = 51)		Medium exposure 2013* (*n* = 287)		High exposure 2013* (*n* = 126)	
	OR (95% CI)^†^	% (*n*/*N*)	OR (95% CI)^†^	% (*n*/*N*)	OR (95% CI)^†^	% (*n*/*N*)	OR (95% CI)^†^	% (*n*/*N*)
Respiratory symptoms	1 (reference)	18.5 (34/184)	1.36 (0.59 to 2.99)	22.0 (11/50)	1.34 (0.82 to 2.22)	23.5 (67/285)	1.27 (0.69 to 2.34)	23.4 (29/124)
			1 (reference)		1.07 (0.53 to 2.30)		1.03 (0.47 to 2.36)	
Stomach pain/nausea	1 (reference)	28.1 (52/185)	0.20 (0.01 to 1.55)	24.5 (12/49)	0.27 (0.01 to 1.77)	28.2 (79/280)	0.21 (0.01 to 1.42)	22.0 (27/123)
			1 (reference)		1.23 (0.62 to 2.60)		0.88 (0.40 to 2.00)	
Anxiety/worries	1 (reference)	24.0 (44/183)	0.75 (0.29 to 1.78)	16.3 (8/49)	1.41 (0.88 to 2.29)	27.2 (77/283)	1.43 (0.77 to 2.63)	24.0 (29/121)
			1 (reference)		1.92 (0.90 to 4.59)		1.70 (0.74 to 4.28)	
Behavioural problems	1 (reference)	18.7 (34/182)	0.54 (0.17 to 1.41)	11.0 (5/47)	1.02 (0.62 to 1.71)	19.0 (53/278)	1.07 (0.57 to 2.00)	18.5 (22/119)
			1 (reference)		1.99 (0.82 to 5.98)		1.98 (0.75 to 6.24)	
Headache(s)	1 (reference)	24.2 (45/186)	0.61 (0.24 to 1.43)	16.3 (8/49)	1.30 (0.81 to 2.08)	27.0 (33/122)	1.38 (0.76 to 2.49)	27.0 (33/122)
			1 (reference)		2.15 (0.98 to 5.25)		2.31 (0.98 to 6.00)	
Depressed mood	1 (reference)	27.3 (50/183)	0.37 (0.13 to 0.90)	12.2 (6/49)	1.06 (0.68 to 1.68)	28.3 (80/283)	0.83 (0.46 to 1.49)	21.3 (26/122)
			1 (reference)		2.82 (1.24 to 7.63)		1.70 (0.74 to 4.28)	
Sleep disturbances	1 (reference)	22.7 (42/185)	0.41 (0.13 to 1.04)	10.4 (5/48)	0.93 (0.58 to 1.51)	22.1 (62/280)	1.10 (0.62 to 1.96)	25.4 (31/122)
			1 (reference)		2.42 (1.00 to 7.23)		2.82 (1.10 to 8.73)	

*Regions are seen in Figure 1

^†^OR and 95% CI from multivariate logistic regression adjusted for age and gender.

## 4. Discussion

The findings from this prospective population-based study indicate that children exposed to a volcanic eruption are more likely to experience respiratory symptoms, anxiety or worries, headache(s) and sleep disturbances, reported by their parents, than children living in a non-exposed region 6–9 months following the eruption. This was reflected in a dose-dependent relationship, with highly exposed children presenting higher likelihood of symptoms than medium or low exposed. Gender stratifications revealed an increased risk of anxiety or worries in both exposed boys and girls whereas only exposed boys, not girls, were more likely to experience headache(s) and sleep disturbances, compared to those who were not exposed to the eruption.

With regard to predictive factors, we found that children who had experienced damage to their homes or other possessions were at increased risk of anxiety or worries and depressed mood compared to those who did not experience such damages. Furthermore, results indicated that exposed parents experiencing psychological morbidity were not more likely to report their children experiencing symptoms than non-exposed parents with psychological morbidity.

None of the observed prevalence of symptoms in the exposed children in 2010 had subsided by 2013 and still then, certain symptoms were associated with severity of exposure, such as depressed mood and sleep disturbances.

### 4.1. Respiratory health

Our findings from 2010, 6–9 months after the Eyjafjallajökull eruption began, show a dose response tendency between levels of exposure to the volcano and respiratory symptoms among the children, as well as adults. This is consistent with previous studies on adults (Yano et al., 1990; Yano, Yokoyama, & Nishii, 1986) and children (Naumova et al., 2007). These results are also in accordance with our previous study on adults which found high exposed adults to be at increased risk of respiratory symptoms, such as shortness of breath, cough and phlegm, compared to a low exposed group 6–9 months after the eruption in Eyjafjallajökull (Carlsen et al., 2012). Long-term adverse health effects on adults have previously been observed after the Eyjafjallajökull eruption, where the risk of some respiratory symptoms increased with time and three years after the eruption, level of exposure continued to be a risk factor for symptom severity (Hlodversdottir et al., 2016). This did not apply to respiratory symptoms among children in our study, as we did not detect any changes in respiratory symptoms between 2010 and 2013 and no difference was found between level of exposure and symptoms by 2013. The fact that respiratory symptoms had not decreased in 2013 remains a concern. Previous studies on long-term respiratory symptoms are scarce. One study we found indicated that children who had ever lived in areas with moderate or heavy exposure to ash on the island Montserrat reported more respiratory symptoms and greater service utilization for respiratory problems compared to low exposed children (Forbes et al., 2003).

### 4.2. Mental health symptoms

In 2010, the exposed children in our study were more likely to show stress related symptoms like anxiety or worries, headache(s), and sleep disturbances than the low or non-exposed, according to their parents reporting about their children´s health. Our results are in line with prior research, which although limited, has found that children and adolescents exposed to disasters commonly have persistent sleep disturbances post-event, both when children and parents report symptomology (Brown, Mellman, Alfano, & Weems, 2011; Dirkzwager, Kerssens, & Yzermans, 2006; Geng, Fan, Mo, Simandl, & Liu, 2013; Usami et al., 2013). To the best of our knowledge, this is the first prospective study assessing sleep disturbances among children following natural disasters. Future research on the population under study should assess the prevalence of PTSD among the children exposed to the eruption as well as the potential influence of PTSD symptoms on the risk of disturbed sleep.

Previous findings have indicated an association between PTSD and other psychological symptoms such as anxiety and depression (Fan et al., 2011; Goenjian et al., 2011; Lai et al., 2015, 2013), higher risks of behavioural problems, substance abuse and sleep disturbance (Pfefferbaum et al., 2015; Usami et al., 2013). This further highlights the importance of studying more diverse symptoms, particularly psychosomatic symptoms.

When comparing girls and boys, previous studies have shown that girls are more likely than boys to develop PTSD symptoms following disaster exposure (Garrison et al., 1995; Giannopoulou et al., 2006; John et al., 2007), although some studies find no such difference (Catani et al., 2008). We did not compare girls and boys regarding prevalence of symptoms, but our gender stratified analyses indicate that both genders show increased risk of symptoms of anxiety or worries but only boys show headache(s) and sleep disturbances.

Highly exposed children in 2010 showed a higher risk of anxiety and/or worries. Our findings also indicate that children whose homes were damaged were more likely to experience anxiety or worries and depressed mood compared to children whose homes were not damaged. Other studies have found disaster experience (both psychological and environmental) to be an important factor in determining a child´s post-disaster mental health (Pfefferbaum et al., 2015; Silverman, 2002; Vernberg, Silverman, La Greca, & Prinstein, 1996).

Our results did not indicate an association between parent’s mental health and children’s symptoms, i.e. parents with symptoms of psychological morbidity reported same prevalence of symptoms among their children as other parents (although heightened but not significant for anxiety or worries and headache(s)). This is not in line with our previous study on childhood avalanche survivors, which found that traumatic reactions of caregivers in the aftermath of the disaster predicted PTSD symptoms among childhood survivors 16 years post-trauma (Thordardottir et al., 2016). In addition, a recent meta-analysis of the association between parental PTSD and/or depressive symptoms and PTSD symptoms in children after exposure to a traumatic event, also confirmed this association (Morris, Gabert-Quillen, & Delahanty, 2012). While our findings are not in coherence with some other studies, this inconsistency further emphasizes the need to include potential moderators in future studies, such as parental well-being.

### 4.3. Health development between 2010–2013

Our findings that children’s symptoms did not subside during the three years following the eruption are surprising. Most previous studies have shown that psychological morbidity decreases over time following natural disasters (Eksi & Braun, 2009; Fan et al., 2015; Piyasil et al., 2011), as also shown in our previous study on adults from the same data collection (Hlodversdottir et al., 2016), while other studies do not show significant changes over time (Jia et al., 2013; Thienkrua et al., 2006; Weems et al., 2010) or they show an increase in symptoms (Shaw et al., 1996; Ye et al., 2014). One reason for our findings might be that proper surveillance and psychological support was not implemented immediately for children after the eruption, indicating that children are a particularly vulnerable group that need developmentally appropriate interventions beyond the needs of adults. Another explanation might be that this is a high-risk area for volcanic eruptions and that the children might fear another and even bigger event, i.e. a volcanic eruption in Katla volcano, located 25 km to the east of Eyjafjallajökull, which is known as one of the most dangerous volcanoes in Iceland (Eliasson, 2014) and has been showing increased seismicity in recent years. This is reflected in a study by Bird and Gísladóttir (2012) which indicates that concerns and media coverage during the Eyjafjallajökull eruption may have increased anxiety among children about the potential hazards of Katla volcano. This might be important and interesting to study.

### 4.4. Strengths and limitations of this study

The main strengths of this study include the ability to clearly define a large population-based cohort and a prospective assessment at two time-points. The high response rates at both points in time minimizes the risk of selection bias. In addition, the exposed population and non-exposed population were similar with regard to background factors. Limitations that should be considered when interpreting the results include that the children were not approached directly and all the measures are based on the reporting of the children´s parents. Parental mental well-being was measured by a valid psychometric measurement but the children’s health was assessed by their parents who answered individual questions on symptoms, but not psychometric questionnaires. Different measurements for adults and reports of children´s parents could therefore explain the discrepancies in results of for example long-term respiratory health effects. Adults were asked in detail about their respiratory health whereas parents received only one question regarding their child’s respiratory health. Also, participants in higher exposed areas may have been more concerned about their children’s health in 2010 but this is not reflected in the differential results found from 2010 and 2013 (dose-response relationship in 2010, but not 2013).

## 5. Conclusions

Natural disasters, such as volcanic eruptions, are threatening events which may cause fear and insecurity, even when there is no loss of life. People may have to evacuate their homes, leave their families and friends and experience severe health challenges. Our study shows that children may present a variety of symptoms, both physical and mental, following such an event that, contrary to previous findings, do not subside over time, underpinning the need for preventative and intervention strategies.
